# Complete Genome Sequence of Noninvasive *Streptococcus pyogenes* M/*emm28* Strain STAB10015, Isolated from a Child with Perianal Dermatitis in French Brittany

**DOI:** 10.1128/genomeA.00806-15

**Published:** 2015-07-16

**Authors:** Suzane de Andrade Barboza, Alexandra Meygret, Pascal Vincent, Séverine Moullec, Nicolas Soriano, Vincent Lagente, Jacques Minet, Samer Kayal, Ahmad Faili

**Affiliations:** a CHU Rennes-Service de Bactériologie et Hygiène Hospitalière, Rennes, France; b Université Rennes 1 Faculté de Médecine, Laboratoire de Microbiologie, Rennes, France; c CNRS, IGDR-UMR 6290, Team GeFMO, Rennes, France; d INSERM, UMR991, Team Remodelage du Microenvironnement, Rennes, France

## Abstract

We report here the complete genome sequence of a noninvasive strain of *Streptococcus pyogenes* M/*emm28*, isolated from perianal dermatitis in a child. The genome is composed of 1,950,454 bp, with a G+C content of 38.2%, and it has 1,925 identified coding sequences and harbors two intact prophages and a new integrating conjugative element (ICE).

## GENOME ANNOUNCEMENT

*Streptococcus pyogenes*, or group A streptococcus (GAS), is a Gram-positive human-specific pathogen that causes a broad range of invasive and noninvasive diseases. Although the throat and skin are the primary ecological niches, the genotype M/*emm28* of GAS has been described as a disease specializing in perineal infections (1). Streptococcal perianal dermatitis (SPD) caused by GAS is a noninvasive infection that occurs mainly in children between 6 months and 5 years of age, affecting boys more often than girls (2). In an effort to gain new insights into invasive M/*emm28* GAS infections, we sequenced and annotated the whole genome of one strain, named STAB10015, isolated in 2010 from a child (a young boy) with SPD and compared it with that of the sequenced strain MGAS6180 isolated from a patient with puerperal sepsis (1).

The strain STAB10015 was grown in Todd-Hewitt medium supplemented with 0.2% yeast extract (THY), and DNA for sequencing was extracted and purified using the phenol-chloroform technique. Genomic DNA was sequenced using HiSeq 2000 technology (Illumina, Inc., San Diego, CA), and the paired-end library was built at the MGX facility of the CNRS in Montpellier, France. There is a total of 36,785,510 high-quality reads, giving an average of 1,933-fold coverage of the genome, which was assembled using the CLC Genomics Workbench version 6 software. The resulting assembly consisted of 56 contigs, which were oriented and connected with the module Microbial Genome Finishing Tools based on the MGAS6180 sequence. After reassembling, 11 gaps persisted, which were filled by PCR, followed by Sanger sequencing. Genome annotation was performed in parallel by using the RAST server (3) and NCBI PGAP (http://ncbi.nlm.nih.gov/genome/annotation_prok). Prophages were identified using the PHAge Search Tool (PHAST) (4). Finally, strain STAB10015 was found to harbor a single circular genome of 1,950,454 bp, with a G+C content of 38.2%. We identified 1,925 coding sequences (CDSs), 65 tRNA genes, 18 rRNA genes, and two intact integrated prophages. The multilocus sequence type (ST) (5) was determined to be ST52.

In comparison with MGAS6180, all known virulence factors (6, 7) (proteinases, gene regulators, and adhesion proteins) were also identified in STAB10015. It contains genes encoding the secreted superantigens SpeC*,* SpeG, SpeJ, SmeZ*,* and SpeK*.* Nonsynonymous single-nucleotide polymorphisms (SNPs) were present in several genes coding for proteases (*speB*), exoenzymes (*hylP*), immunoreactive antigens (*isp1*), and adhesion proteins (*sof*, *enn*, and *spr28* from the region-deleted 2 [RD2] region). Many genes encoding adhesins (*emm*, *mrp-emm*-like, *sclA*, *sclB*, and *sbfx*) or proteases (*scp*) presented significant deletions. We also identified a new integrating conjugative element (ICE) with a length of 54.2 kb, not encoding apparent virulence factors or antibiotic resistance genes, and it is inserted in the 3′ end of the tRNA uracil-5-methyltransferase gene, described as a hot spot for ICE (8). Sequence comparisons indicated significant homology with other intestinal bacterial ICEs (*Streptococcus dysgalactiae* RE378 [9] and *Clostridium difficile* 630 [10]). These bacterial interactions might result in antibiotic resistance gene [*erm*(TR)-*tet*(O)] acquisition, as described for ICE*Sp2905* in the IB21 GAS strain (11).

Before deciphering the bacterial phenotype and invasive potential, it is necessary to compare the STAB10015 sequence with that of strains isolated in the same geographical and temporal context.

### Nucleotide sequence accession numbers.

The complete genome sequence of *S. pyogenes* strain STAB10015 has been deposited in the NCBI under the accession no. CP011068, part of BioProject PRJNA278400.

## References

[1] Green NM, Zhang S, Porcella SF, Nagiec MJ, Barbian KD, Beres SB, LeFebvre RB, Musser JM 2005 Genome sequence of a serotype M28 strain of group a streptococcus: potential new insights into puerperal sepsis and bacterial disease specificity. J Infect Dis 192:760–770. doi:10.1086/430618. 16088825

[2] Amren DP, Anderson AS, Wannamaker LW 1966 Perianal cellulitis associated with group A streptococci. Am J Dis Child 112:546–552. doi:10.1001/archpedi.1966.02090150090007. 5928818

[3] Aziz RK, Bartels D, Best AA, DeJongh M, Disz T, Edwards RA, Formsma K, Gerdes S, Glass EM, Kubal M, Meyer F, Olsen GJ, Olson R, Osterman AL, Overbeek RA, McNeil LK, Paarmann D, Paczian T, Parrello B, Pusch GD, Reich C, Stevens R, Vassieva O, Vonstein V, Wilke A, Zagnitko O 2008 The RAST server: Rapid Annotations using Subsystems Technology. BMC Genomics 9:75. doi:10.1186/1471-2164-9-75. 18261238 PMC2265698

[4] Zhou Y, Liang Y, Lynch KH, Dennis JJ, Wishart DS 2011 PHAST: a fast phage search tool. Nucleic Acids Res 39:W347–W352. doi:10.1093/nar/gkr485. 21672955 PMC3125810

[5] Enright MC, Spratt BG, Kalia A, Cross JH, Bessen DE 2001 Multilocus sequence typing of *Streptococcus pyogenes* and the relationships between *emm* type and clone. Infect Immun 69:2416–2427. doi:10.1128/IAI.69.4.2416-2427.2001. 11254602 PMC98174

[6] Chen L, Yang J, Yu J, Yao Z, Sun L, Shen Y, Jin Q 2005 VFDB: a reference database for bacterial virulence factors. Nucleic Acids Res 33:D325–D328. doi:10.1093/nar/gki008. 15608208 PMC539962

[7] Nakagawa I, Kurokawa K, Yamashita A, Nakata M, Tomiyasu Y, Okahashi N, Kawabata S, Yamazaki K, Shiba T, Yasunaga T, Hayashi H, Hattori M, Hamada S 2003 Genome sequence of an M3 strain of *Streptococcus pyogenes* reveals a large-scale genomic rearrangement in invasive strains and new insights into phage evolution. Genome Res 13:1042–1055. doi:10.1101/gr.1096703. 12799345 PMC403657

[8] Brenciani A, Tiberi E, Bacciaglia A, Petrelli D, Varaldo PE, Giovanetti E 2011 Two distinct genetic elements are responsible for *erm*(TR)-mediated erythromycin resistance in tetracycline-susceptible and tetracycline-resistant strains of *Streptococcus pyogenes*. Antimicrob Agents Chemother 55:2106–2112. doi:10.1128/AAC.01378-10. 21343455 PMC3088260

[9] Okumura K, Shimomura Y, Murayama SY, Yagi J, Ubukata K, Kirikae T, Miyoshi-Akiyama T 2012 Evolutionary paths of streptococcal and staphylococcal superantigens. BMC Genomics 13:404. doi:10.1186/1471-2164-13-404. 22900646 PMC3538662

[10] Sebaihia M, Wren BW, Mullany P, Fairweather NF, Minton N, Stabler R, Thomson NR, Roberts AP, Cerdeño-Tárraga AM, Wang H, Holden MT, Wright A, Churcher C, Quail MA, Baker S, Bason N, Brooks K, Chillingworth T, Cronin A, Davis P, Dowd L, Fraser A, Feltwell T, Hance Z, Holroyd S, Jagels K, Moule S, Mungall K, Price C, Rabbinowitsch E, Sharp S, Simmonds M, Stevens K, Unwin L, Whithead S, Dupuy B, Dougan G, Barrell B, Parkhill J 2006 The multidrug-resistant human pathogen *Clostridium difficile* has a highly mobile, mosaic genome. Nat Genet 38:779–786. doi:10.1038/ng1830. 16804543

[11] Giovanetti E, Brenciani A, Tiberi E, Bacciaglia A, Varaldo PE 2012 ICE*Sp2905*, the *erm*(TR)-*tet*(O) element of *Streptococcus pyogenes*, is formed by two independent integrative and conjugative elements. Antimicrob Agents Chemother 56:591–594. doi:10.1128/AAC.05352-11. 21986826 PMC3256084

